# Association between Immunoglobulin G Levels and Adverse Effects Following Vaccination with the BNT162b2 Vaccine among Japanese Healthcare Workers

**DOI:** 10.3390/vaccines9101149

**Published:** 2021-10-09

**Authors:** Jun Otani, Ryuichi Ohta, Chiaki Sano

**Affiliations:** 1 Community Care, Unnan City Hospital, Daito-cho, Shimane Prefecture, Unnan 699-1221, Japan; blackjack.otani@nifty.ne.jp; 2 Department of Community Medicine Management, Faculty of Medicine, Shimane University, Shimane Prefecture, Izumo 693-8501, Japan; sanochi@med.shimane-u.ac.jp

**Keywords:** BNT162b2 vaccine, adverse reactions, immunologic response

## Abstract

The purpose of the study was to assess the association between the amount of immunoglobulin G (IgG) and the duration of adverse effects of COVID-19 vaccinations in the Japanese population. This cross-sectional study was conducted from April 2020 to August 2021 among workers at a community hospital. All participants received two doses of the BNT162b2 vaccine (Pfizer-BioNTech) in March and April 2021. Vaccine side effects were measured using a self-administered questionnaire. Serum anti-SARS-CoV-2 IgG was measured 3 months after vaccination. There was a total of 338 participants (mean age: 44.7 years). The incidence of adverse reactions after vaccination was higher in women. Adverse reactions associated with higher IgG levels included: erythema at the injection site after the first dose; induration and inflammation at the injection site; and systemic symptoms, e.g., fever and headache after the second dose. IgG levels were higher in younger participants. These findings could mitigate fears regarding the mild adverse effects of the COVID-19 vaccine and encourage uptake of the BNT162b2 vaccine.

## 1. Introduction

The COVID-19 pandemic has had an impact on people’s lives. The global spread of COVID-19 has killed many people worldwide and inhibited the movement of people outside their homes and districts [1,2]. Various infection control measures, such as lockdowns and states of emergency, have been implemented to prevent the spread of COVID-19 [3,4]. Various infection control campaigns, such as handwashing and avoiding places where there is a high risk of infection, have also been implemented [5,6]. However, these measures have not terminated the pandemic and have led to intermittent waves of infection [7,8]. Currently, COVID-19 vaccination is one of the prevention measures that has the potential to control the pandemic [9]. After the introduction of the first COVID-19 vaccine by Pfizer and BioNTech in December 2020, various manufacturers developed vaccines and distributed them worldwide [10]. Although the effectiveness of the vaccines varies, they provide a reasonable level of protection against COVID-19 and reduce the mortality rate [11,12]. A high level of vaccine coverage has the potential to stop the COVID-19 pandemic, so vaccination is being encouraged worldwide [13].

One of the problems associated with vaccination is a vaccine’s potentially adverse effects. COVID-19 vaccines were developed rapidly for general use, and various side effects have been reported worldwide [14,15]. The rate of adverse effects of COVID-19 vaccines differs between countries because the type of vaccines used also differs by country [16]. Adverse effects range from mild to severe, including localized reactions, such as swelling and a rash at the injection site, and systemic reactions, including dyspnea and anaphylaxis [12,17]. Therefore, many people who do not have a general phobia toward vaccines tend to avoid vaccination because of a fear of adverse effects influenced by social media. Low vaccination coverage can inhibit the establishment of herd immunity at a community level [18,19]. The severity and duration of adverse vaccine reactions varies and is correlated with the serum immunoglobulin G (IgG) level [20]. As people generally desire to be immunized against COVID-19, clarification of the association between the intensity of the immune response toward COVID-19 vaccines and the types of adverse effects could motivate people to be vaccinated and endure the adverse effects of vaccination.

Clarification of the association between the intensity of the immune response against COVID-19 and the types of adverse effects according to recipient characteristics is critical for mitigating the fear of COVID-19 vaccination and regaining a safer world with herd immunity. However, there is currently limited information available on the association between the level of anti-SARS-CoV-2 IgG and the types of adverse effects of COVID-19 vaccination. Reactions to vaccines depend not only on the type of vaccine but also on individual characteristics [21,22]. In this study, we focused on a Japanese population that had received the Pfizer-BioNTech BNT162b2 vaccine. The purpose of this study was to clarify the association between the IgG response, the types of adverse effects, and the recipients’ characteristics in the Japanese population.

## 2. Materials and Methods

### 2.1. Design

This cross-sectional study was conducted among staff who worked at a community hospital between April 2020 and August 2021.

### 2.2. Setting

Unnan City is one of the most rural cities in Japan and is located in the southeast of Shimane Prefecture. In 2020, the total population of Unnan was 37,638 (18,145 were male and 19,492 were female), with 39% aged over 65 years. It is expected that 50% of the population will be aged over 65 years by 2025 [23]. This city has one of the lowest numbers of physicians per 1000 people in Japan. There are 16 clinics, 12 home care stations, 3 visiting nurse stations, and 1 public hospital (Unnan City Hospital). The hospital staff comprises 27 physicians, 197 nurses, 7 pharmacists, 15 clinical technicians, 37 therapists (22 physical therapists, 12 occupational therapists, and 3 speech therapists), 4 nutritionists, and 34 clerks. The hospital has departments of general internal medicine, surgery, orthopedics, pediatrics, dermatology, urology, otolaryngology, obstetrics and gynecology, and general medicine, run by full-time doctors. There are no other medical institutions with recovery rehabilitation units in the city [24].

### 2.3. Participants

The study participants were healthcare workers who worked at a community hospital from April 2020 to August 2021. All participants were vaccinated with two doses of the BNT162b2 vaccine in March and April 2021.

### 2.4. Measurements

#### Demographic Data of Patients

Data were collected on participant demographic characteristics such as age, sex, and occupation. Adverse effects were measured twice, once after each dose of vaccine, using a self-administered questionnaire on symptoms related to the vaccination. The list of adverse effects consisted of local reactions at the injection site (induration, heat, itchiness, pain, redness, and swelling) and systemic symptoms (fatigue, fever, headache, and rhinorrhea). 

Serum IgG levels against SARS-CoV-2 were measured 3 months after the second vaccination using the SARS-CoV-2 IgG II Quant Reagent Kit (Abbott). 

### 2.5. Statistical Analysis

The Student’s *t*-test was performed on parametric data, and the Mann–Whitney U test was performed on non-parametric data regarding background data, adverse effects, and IgG level. The correlations between the IgG level, the occurrence of adverse effects, and participant characteristics were analyzed using chi-squared tests and Spearman correlation (r). Regarding the sample size calculation, it was estimated that 126 participants would be required for 80% statistical power and a 5% chance of a type I error to detect a correlation between the occurrence of adverse effects and the IgG level. Participants with missing data were excluded from the analysis. Statistical significance was defined as a *p*-value < 0.05. All statistical analyses were performed using EZR (Saitama Medical Center, Jichi Medical University, Saitama, Japan), which is a graphical user interface for R (The R Foundation, Vienna, Austria) [25].

### 2.6. Ethics Approval

The Unnan City Hospital Clinical Ethics Committee approved this study (approval number: 20210012). All participants provided informed consent, and research information was posted on the hospital website without any identifying details. Contact information for the hospital representative was also listed on the website. All procedures included in this study were performed in compliance with the Declaration of Helsinki and its subsequent amendments.

## 3. Results

### 3.1. The Demographic Data of the Participants

**Figure 1:**
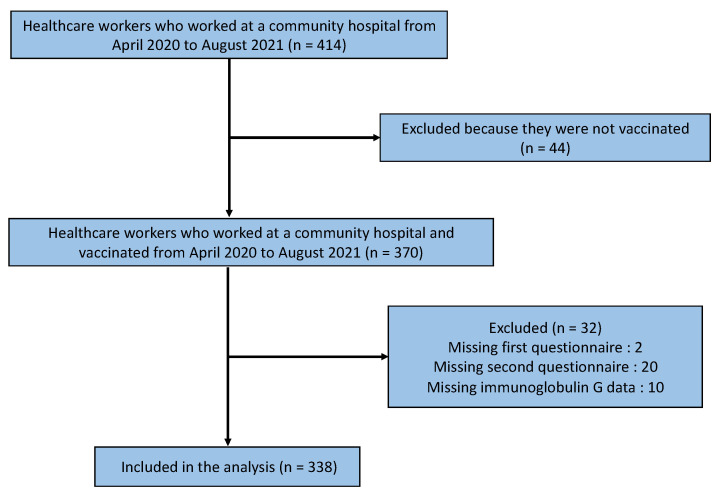
Flowchart of the participant selection process.

Figure 1 shows a flowchart of the participant selection process. A total of 414 healthcare workers worked at the hospital from April 2020 to August 2021, of whom 370 were vaccinated between April 2020 and August 2021. Of these, 32 participants were excluded due to a lack of data on the first and second questionnaires or their IgG levels, leaving 338 participants in the analysis (Figure 1).

The mean age of participants was 44.7 years (standard deviation: 12.5 years), of whom 72.4% (239/338) were female. None of the participants experienced critical adverse reactions. The job category (*p* < 0.001), incidence of symptoms after the first (*p* = 0.026) and second (*p* = 0.007) doses of the vaccine, and the incidence of itching (*p* = 0.015), fatigue (*p* = 0.002), headache (*p* = 0.026), and rhinorrhea (*p* = 0.011) after the first dose; and itching (*p* < 0.001), redness (*p* = 0.038), fatigue (*p* = 0.015), and headache (*p* = 0.003) after the second dose, differed significantly by sex (Table 1).

### 3.2. The Relationship between Adverse Effects, the Backgrounds, and the Amount of IgG

**Figure 2:**
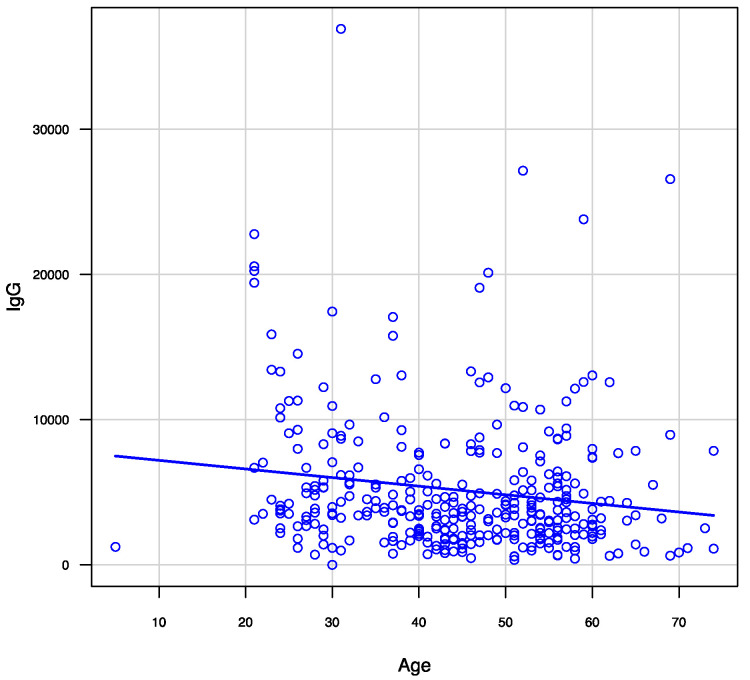
Correlation between age and anti-SARS-CoV-2 IgG levels 3 months after receiving two doses of the BNT162b2 vaccine.

Regarding the amount of IgG against COVID-19, redness on the injection site after the first dose (*p* = 0.025), hardness (*p* = 0.027), heat (*p* < 0.001), swelling (*p* = 0.018) on the injection site, and systemic symptoms (fatigue [*p* < 0.001], fever [*p* = 0.003], and headache [*p* < 0.001]) after the second dose, were associated with a higher amount of IgG against COVID-19 (Table 2). Regarding the relationship between age and the amount of IgG against COVID-19, there was a negative correlation (Spearman r = −0.167, *p* = 0.002) (Figure 2).

## 4. Discussion

This study shows that there was an association between the vaccine recipients’ age and sex in the incidence of adverse reactions and IgG response after receiving the BNT162b2 vaccine. The incidence of adverse reactions after the vaccine was higher in women than in men. Adverse reactions associated with higher IgG levels included redness at the injection site after the first dose and induration, heat, and swelling at the injection site, along with systemic symptoms, fever, and headache after the second dose. IgG levels were inversely related to age.

The difference in the incidence of adverse reactions according to sex may have been related to immunological differences in response to vaccination according to sex. Vaccines stimulate an artificial immunological response, leading to the production of specific antibodies against specific antigens [26]. This process occurs through the adaptive immune system [27]. The strength of the adaptive immune system differs according to sex [28,29]. Women tend to have a stronger reaction in immunological activation, leading to a higher incidence of autoimmune disease than men [30]. In this study, female participants had a higher incidence of adverse reactions than male participants after the first and second doses of the vaccine. These reactions include itching, fatigue, headache, and rhinorrhea after the first dose and itching, redness, fatigue, and headache after the second dose. These findings are consistent with those reported in previous studies on other vaccines [31,32,33]. Although none of the participants experienced serious adverse events following vaccination, care should be taken to monitor women for adverse effects after receiving the COVID-19 vaccination. 

The difference in anti-SARS-CoV-2 IgG levels, according to demographic characteristics and adverse reactions, may be related to vaccine recipients acquiring immunity against SARS-CoV-2. This study showed that redness at the injection site after the first dose, induration, heat, swelling at the injection site, and systemic symptoms (fatigue, fever, and headache) after the second dose were associated with a higher level of anti-SARS-CoV-2 IgGV. Redness at the injection site after the first dose was associated with a strong immunological reaction, which may be related to a strong reaction to the first exposure to a foreign antigen [33,34]. The lack of difference in IgG level according to the incidence of redness at the injection sites following the second dose of vaccine may be because of an adaptive immunological reaction. A strong reaction to the first dose of the vaccine, such as redness at the injection site, may be associated with acquiring a higher level of immunity against SARS-CoV-2. The second dose of the vaccine induced a stronger inflammatory reaction at the injection site, and a strong reaction to the second dose was associated with a higher IgG level, which suggests that the inflammatory reaction to the second dose of vaccine may be related to acquiring stronger immunity against SARS-CoV-2 [35]. Furthermore, after the second dose, systemic symptoms such as fatigue, fever, and headache can be triggered by immunological reactions [36]. After the first dose, the recipients had stimulated lymphocytes as a result of an adaptive immunological reaction. The second dose can trigger systemic inflammation, and recipients with strong immunity are more likely to experience systemic symptoms. Previous studies have reported the association between the presence of adverse reactions and the IgG titer [33,37]. However, they did not clearly show the association between concrete adverse effects and IgG titers. This study investigated the presence of the specific adverse symptoms associated with high IgG titers.

Age may also be a factor in the acquisition of immunity following COVID-19 vaccination. This study showed that age was correlated with the amount of anti-SARS-CoV-2 IgG and that IgG levels were higher in younger participants. This may be related to the viability of immunity in the younger population compared with that of older populations, which has been shown in previous studies on other vaccines [37,38]. Younger individuals tend to have more T and B cells related to adaptive immunity and active toll-like receptor reactions, which have a greater association with higher IgG levels among younger individuals than older individuals [37,39]. Currently, the incidence of COVID-19 is increasing among younger individuals worldwide. Younger individuals spread SARS-CoV-2 infection because of their activity and interactivity [40]. As their immunity can effectively be modified by vaccination, these results suggest a need to focus on vaccinating younger individuals.

These results provide information on the incidence of adverse reactions, which can inform policy. Vaccine hesitancy is a critical challenge to attaining high COVID-19 vaccine coverage [19,41]. One of the causes of vaccine hesitancy may be the adverse effects of vaccination [42]. Based on previous research, adverse effects impede the uptake of vaccination in communities [43]. The COVID-19 pandemic has disrupted human activities. One of the ways of normalizing this abnormal situation is COVID-19 vaccination. People hope to acquire immunity against SARS-CoV-2 infection [44]. This study has shown a relationship between the strength of adverse effects and the immune response. Providing information about the relationship between adverse effects and the acquisition of immunity may help individuals overcome their fear of adverse effects, making them more willing to endure adverse effects in order to acquire strong immunity against SARS-CoV-2 infection [45,46]. For the effective distribution of the COVID-19 vaccine during the pandemic, appropriate information should be provided about the relationship between the intensity of adverse effects and the amount of acquired immunity against COVID-19. Effective sharing of difficulties and providing healthcare information regarding the COVID-19 vaccine among citizens can improve health literacy, relationships, and satisfaction among community members, especially in rural areas, during the pandemic [47,48]. To sustain rural communities, citizens and central and local governments should collaborate to provide appropriate information regarding COVID-19 vaccines.

One of the limitations of this study was its small sample size. In order to provide generalized results, larger studies with more diverse participants are required. This study can serve as a facilitator for future studies. Furthermore, this study lacked a comparison group including individuals who did not undergo vaccination and a range of follow-up periods; therefore, we could not show cause-and-effect relationships. In this study, we used a self-report questionnaire to document the limited adverse effects of the vaccine without the scale of the effects, which may have reduced the internal validity of the results. Future studies should use observers to monitor the adverse effects of vaccination to improve internal validity.

## 5. Conclusions

This study showed an association between demographic characteristics, adverse reactions, and the immunological response to the COVID-19 vaccine. The incidence of adverse reactions after receiving the COVID-19 vaccination was higher in women than in men. Some adverse reactions were associated with a higher IgG level, of which the younger participants were more susceptible. These findings can be used to alter perceptions about the adverse effects of vaccination and encourage more individuals to undergo COVID-19 vaccination.

## Figures and Tables

**Table 1:** Incidence of symptoms after vaccination with the BNT162b2 vaccine according to sex.

Factor	Overall n = 338	Sex		*p* Value ^1^
		Female n = 239	Male n = 99	
**Age**	44.7 (12.5)	45.4 (12.4)	42.9 (12.8)	0.099
Job (%)				
Physician	23 (6.8)	2 (0.8)	21 (21.2)	<0.001
Nurse	215 (63.6)	198 (82.8)	17 (17.2)	
Other medical staff	76 (22.5)	31 (13.0)	45 (45.5)	
Clerk	24 (7.1)	8 (3.3)	16 (16.2)	
IgG against COVID-19 ^2^	5147 (4708)	5231 (4909)	4944 (4200)	0.611 ^3^
**First dose**				
Symptoms				0.026
+	298 (88.2)	217 (90.8)	81 (81.8)	
−	40 (11.8)	22 (9.2)	18 (18.2)	
**Injection site**				
Induration				0.849
+	37 (10.9)	27 (11.3)	10 (10.1)	
−	301 (89.1)	212 (88.7)	89 (89.9)	
Heat				0.736
+	49 (14.5)	36 (15.1)	13 (13.1)	
−	289 (85.5)	203 (84.9)	86 (86.9)	
Itching				0.015
+	40 (11.8)	35 (14.6)	5 (5.1)	
−	298 (88.2)	204 (85.4)	94 (94.9)	
Pain				0.103
+	284 (84.0)	206 (86.2)	78 (78.8)	
−	54 (16.0)	33 (13.8)	21 (21.2)	
Redness				0.654
+	26 (7.7)	20 (8.4)	6 (6.1)	
−	312 (92.3)	219 (91.6)	93 (93.9)	
Swelling				0.507
+	51 (15.1)	34 (14.2)	17 (17.2)	
−	287 (84.9)	205 (85.8)	82 (82.8)	
**Systemic symptoms**				
Fatigue				0.002
+	61 (18.0)	53 (22.2)	8 (8.1)	
−	277 (82.0)	186 (77.8)	91 (91.9)	
Fever				0.559
+	3 (0.9)	3 (1.3)	0 (0.0)	
−	335 (99.1)	236 (98.7)	99 (100.0)	
Headache				<0.001
+	53 (15.7)	49 (20.5)	4 (4.0)	
−	285 (84.3)	190 (79.5)	95 (96.0)	
Rhinorrhea				0.011
+	20 (5.9)	19 (7.9)	1 (1.0)	
−	318 (94.1)	220 (92.1)	98 (99.0)	
**Second dose**				
Symptoms				0.007
+	326 (96.4)	235 (98.3)	91 (91.9)	
−	12 (3.6)	4 (1.7)	8 (8.1)	
**Injection site**				
Induration				0.162
+	61 (18.0)	48 (20.1)	13 (13.1)	
−	277 (82.0)	191 (79.9)	86 (86.9)	
Heat				0.159
+	109 (32.2)	83 (34.7)	26 (26.3)	
−	229 (67.8)	156 (65.3)	73 (73.7)	
Itching				<0.001
+	78 (23.1)	71 (29.7)	7 (7.1)	
−	260 (76.9)	168 (70.3)	92(92.9)	
Pain				0.181
+	302 (89.3)	217 (90.8)	85 (85.9)	
−	36 (10.7)	22 (9.2)	14 (14.1)	
Redness				0.038
+	69 (20.4)	56 (23.4)	13 (13.1)	
−	269 (79.6)	183 (76.6)	86 (86.9)	
Swelling				0.067
+	100 (29.6)	78 (32.6)	22 (22.2)	
−	238 (70.4)	161 (67.4)	77 (77.8)	
**Systemic symptoms**				
Fatigue				0.015
+	231 (68.3)	173 (72.4)	58 (58.6)	
−	107 (31.7)	66 (27.6)	41 (41.4)	
Fever				0.575
+	79 (23.4)	58 (24.3)	21 (21.2)	
−	259 (76.6)	181 (75.7)	78 (78.8)	
Headache				0.003
+	167 (49.4)	131 (54.8)	36 (36.4)	
−	171 (50.6)	108 (45.2)	63 (63.6)	
Rhinorrhea				0.343
+	38 (11.3)	30 (12.6)	8 (8.2)	
−	299 (88.7)	209 (87.4)	90 (91.8)	

The values are n (%) unless otherwise indicated. ^1^ *p*-values were calculated using chi-squared tests unless otherwise indicated. ^2^ The values shown are the mean and standard deviation. ^3^ The *p*-value was calculated using Student’s *t*-test and chi-squared test.

**Table 2:** Anti-SARS-CoV-2 IgG levels at 3 months after receiving two doses of the BNT162b2 vaccine according to sex and vaccine-associated symptoms.

Factor	Min	25%	Median	75%	Max	*p* Value
Sex						0.664
Male	0	2068.6	3599.5	6427.8	23,799	
Female	415.8	2326.6	3819.3	6159.9	36,908.4	
**First dose**						
Symptoms						0.340
+	0	2365.8	3764.7	6177.6	36,908.4	
−	583	2095.5	3425.35	6323.0	23,799	
**Injection site**						
induration						0.114
+	0	3337.3	4506.8	7119.2	20,556.3	
−	338.1	2186.5	3599.1	6148.2	36,908.4	
Heat						0.346
+	0	2049.9	4502.8	6679.1	36,908.4	
−	338.1	2203	3619.9	6148.2	27,150.8	
Itching						0.921
+	0	2415.5	3398.7	6915.4	27,150.8	
−	338.1	2191.9	3717.0	6165.7	36,908.4	
Pain						0.373
+	0	2369.3	3792.8	6281.9	36,908.4	
−	583	2029.5	3600.4	5593.6	27,150.8	
Redness						0.0247
+	0	3636.8	5431.3	7860.2	15,870.6	
−	338.1	2189.4	3600.4	6040.2	36,908.4	
Swelling						0.146
+	0	2101.9	4502.8	7623.3	20,556.3	
−	338.1	2207.8	3599.5	5817.9	36,908.4	
**Systemic symptom**						
Fatigue						0.233
+	415.8	2491.9	4335.4	6701.7	15,771.6	
−	0	2128.5	3642.4	6018.8	36,908.4	
Fever						0.386
+	415.8	6729.4	13,042.9	16,799.6	20,556.3	
−	0	2197.9	3678.2	6159.9	36,908.4	
Headache						0.826
+	697.7	2437.9	3973.2	6104.3	20,556.3	
−	0	2192.8	3655.8	6179.6	36,908.4	
Rhinorrhea						0.776
+	779.5	2175.7	4167.9	5204.6	13,439.6	
−	0	2195.35	3667	6230.1	36,908.4	
**Second dose**						
Symptoms						0.537
+	0	2208.0	3748.1	6165.7	36,908.4	
−	467.4	2055.6	2920.55	6401.1	10,958	
**Injection site**						
Induration						0.0266
+	0	3063.3	4666.8	7905.3	19,431.2	
−	338.1	2186.5	3519.9	5972.4	36,908.4	
Heat						<0.001
+	0	2538.7	4666.8	8308.6	27,150.8	
−	338.1	2128.5	3444.1	5430.6	36,908.4	
Itching						0.306
+	415.8	2827.4	4003.3	6154.7	27,150.8	
−	0	2173.0	3600.4	6196.4	36,908.4	
Pain						0.078
+	0	2377.8	3826.1	6351.75	36,908.4	
−	467.4	1780.0	2920.6	5176.1	17,446	
Redness						0.527
+	415.8	2506.4	4283.7	5600.5	22,778.1	
−	0	2186.5	3601.3	6386.7	36,908.4	
Swelling						0.018
+	0	2217.9	4571.9	7861.2	27,150.8	
−	338.1	2188.1	3513.6	5644.5	36,908.4	
**Systemic symptoms**						
Fatigue						<0.001
+	0	2460.9	4151.3	7545.5	36,908.4	
−	338.1	1762.7	3045.1	4500.9	15,771.6	
Fever						0.0025
+	0	2587.2	5132.3	8705.7	36,908.4	
−	338.1	2160.4	3515.4	5528.7	22,778.1	
Headache						<0.001
+	0	2512.7	4344.4	7711.7	36,908.4	
−	338.1	1858.9	3405	5481.2	26,561.4	
Rhinorrhea						0.323
+	765.5	2820.9	4365.5	5595.5	20,556.3	
−	0	2188.4	3619.9	6213.3	36,908.4	

## Data Availability

The datasets used and/or analyzed during the current study are available from the corresponding author upon reasonable request.
